# Alcohol Consumption by Russian Workers Before and During the Economical Reforms of the 1990s

**DOI:** 10.5812/ijhrba.11004

**Published:** 2013-09-20

**Authors:** Sergei V Jargin

**Affiliations:** 1 Department of Public Health, Peoples’ Friendship University of Russia, Moscow, Russia

**Keywords:** Alcoholism, Life Expectancy, Russia, Wine

Consumption of surrogates was indirectly stimulated with certain anti-alcohol measurments taken by government Soviet-time, sorts of cheap fortified wine, was largely disappeared during the 1990s; however wine surrogates were continuously sold out. Authorities should take measurments to prevent the sales of poor-quality and counterfeit alcoholic beverages. In conclusion, some policies contributed to the mortality increase among workers and some other social groups. Apart from alcohol, limited modern health care is another obvious reason of the relatively low life expectancy in Russia.

Alcohol consumption in the former Soviet Union (SU) rapidly increased after the anti-alcohol campaign (1985-88). At the same time, there was an increase about unrecorded consumption (1). The economic reforms with privatization of many factories during the 1990s occurred in conditions of massive alcohol consumption by workers. The fact that the state at various times, encouraged alcohol sales is known to the international community (2). Retrospectively, it becomes clear for inside observers that the anti-alcohol campaign (1985-88) was just another tool used for the same purpose, with a predictable failure and a recoil effect at the required moment. Alcohol abusers can experience emotions of shame, guilt and have a low self-esteem (3); therefore probably easier being to manipulate and command. Drunkenness, truancy, and petty larceny at workplaces were often tolerated with the management at that and earlier time. In a sense, it facilitated the economic reforms. Many workers did not actively oppose privatizations, performed not always in accordance with the law, because they had not been innocent themselves. 

*Anti-alcohol Measurements and Alcohol Consumption*

Anti-alcohol measurments were taken by the government also before the campaign of 1985-88. In 1972, it was prohibited to sell vodka before 11 a.m. to after 7 pm. and on Sundays. However, after 7 pm. till the closing of shops at 8-10 pm. different sorts of cheap fortified wine were available (alcohol volume 17-19%). Besides, vodka was sold not in all shops. As a result, many workers, finished their work around 5 pm., considering queues at shops to drink a dose of vodka (often still at the workplace) but continued with fortified wine or beer. Some workers went to a beerhouse, often having with them a bottle of vodka for 2-3 persons. However, beerhouses were not numerous at that time and often overcrowded. Beer usually contained 3-4% volume of alcohol and was suspected to be additionally diluted at beerhouses; so that many people preferred to consume stronger beverages. Quality of fortified wine was uneven; cheaper varieties often had poorer lower quality. It was generally known by drinking public that it is not advisable to drink fortified wine after vodka: it often ended up with severe intoxication, vomiting, loss of self-control, falling asleep in public places, detentions by militia (police) etc.

Furthermore, the 0.25 l vodka bottles (chetvertinka) largely disappeared in the early 1970s; and predominantly 0. 5l bottles were sold. Aged alcoholics said that, when they went home after work, having already drunk 1-2 glasses of vodka (under a "glass" a 250 ml glass was generally understood), it would be optimal for them to buy a 0.25 l bottle of vodka: then they would safely come home and wake up in the morning fit for the next working day. Instead, they continued with fortified wine, which caused, as mentioned above, a deeper intoxication, associated with different risks and severe hangover next morning. The cheapest fortified wine was named in popular language "bormotukha" - the "mumbler". It acted stupefying; intoxicated individuals sometimes mumbled indeed and often lost control over their speech and actions. Whether it was caused by poor quality alcohol i.e. compounds other than ethanol occurring naturally in alcohol beverages (4) or by some additives. It is obviously easier to add substances to those red or brownish fluids with different tastes and flavors, than to a rather standard product such as vodka. Workers of wine-making industry, with whom I spoke about it, witnessed that the substance named "bentonite" (pronounced by them like "betonit") was added to cheap wines; probably it was just sorbent. Under the conditions described above, there was often no other choice for alcohol abusers than to start with vodka and continue with fortified wine or wine surrogate (or to consume the latter only). Certainly, there is always a choice to drink less or not to drink at all; however, the life during the late Soviet period was rather monotonous, and there were not many alternative pastimes available for workers, especially middle-aged ones, living for their salaries. Paradoxically, more choices had those, who purloined goods at workplace, used the factory's equipment, or trucks in case of drivers, for side-line works, or had other illegal incomes: such people could afford the purchase of cars, garages, summer cottages (dachas) etc., which provided pastime opportunities without alcohol consumption.

*Consequences for Public Health*

By 1993, the average expectation of life for Russian men was estimated to be 59 years old (5). In 2008, the difference in life expectancy between men in some West European countries and Russia was reported to be 20 years (6). Base on my research on pathology in Russia and abroad, pulmonary diseases (chronic exacerbating bronchitis, pneumonia etc.) have been a more frequent reason for death of alcohol abusers in Russia, which partly explained with climate and lesser availability of warm public houses, especially for working class and pauper drinkers; some of them did not live long enough to die from liver cirrhosis. Possibility of exogenous immunodeficiency might be considered as well. The relatively high mortality rates from cardiovascular diseases in Russia during 1990s compared to other countries (6, 7) were commented in (8): since the Soviet time, autopsy has remained obligatory for the patients dying in hospitals, but the attitude has become more formal and superficial. Post mortem examinations have often been performed incompletely, without much insight. If a cause of death is not entirely clear, it is usual to write in death certificate “ischemic heart disease with cardiac insufficiency” or a similar formulation. There has been a tendency to over diagnose cardiovascular diseases in unclear cases also among people dying at home and not undergoing autopsy. On my opinion, the causative role of alcohol in the relatively high mortality in Russia has been exaggerated e.g. in (9) possibly to veil another important cause: limited access to the modern health care especially for middle-aged and elderly men (8). A large number of investigations have given support to the idea that consumption of moderate amounts of alcoholic beverages, particularly wine, protects against coronary heart disease. At the same time, alcohol has several potential adverse effects on the cardiovascular system (10). The cause-effect relationship between alcohol and cardiovascular diseases is not discussed here; it might be only mentioned that some pathologists in the former SU, having experience of autopsy including many alcohol abusers, noticed that alcoholics often have post mortem no atherosclerotic plaques in their arteries. It may be caused not (only) by alcohol itself but also by poor diets of many alcohol abusers. In the literature, a complex relationship between alcoholism and atherosclerosis at autopsy was reported, e.g. that alcoholic men and old women had a significantly lower degree of atherosclerosis in the coronary arteries, while the opposite was found in younger women (11). Obviously, relationships between different patterns of alcohol consumption and cardiovascular morbidity/mortality deserve a separate review and further research by long-term cohort studies (12) under unbiased conditions. Animal experiments might be helpful as well. Finally, evaluating results of surveys (e.g. 13,14), it should be taken into account that surveys and questionnaires are largely discredited in Russia by means of obtrusive solicitations to partake in different surveys, often asking for private information, in the streets, per telephone, and previously also by agents coming to private homes. Answers to the questionnaires can be biased, especially with regard to such a delicate topic as alcohol use and misuse.

Some cheaper fortified wines and wine surrogates were in fact more harmful than vodka sold concomitantly in the former SU during the 1970-90s. As described above, consumption of the wine surrogates with or without vodka was indirectly stimulated by certain anti-alcohol measures. Apparently, there is a tendency to veil these facts in some publications, stressing the harmful properties of vodka as opposed to wine (e.g., 15). It was reported, for example, that of the three beverages (vodka, wine, beer), vodka alone was associated with cardiovascular mortality (7), which appears questionable because the period 1970-2005 was analyzed, including the time when fortified wines were massively sold and consumed. Wine surrogates and fortified wines, containing added distilled spirit with poor quality, are not mentioned in this and some analogous reports. Considering the above and previously published (8, 13) arguments, and certain policies contributed to the mortality increase among workers and some other social groups. Some people name it genocide; certainly, it is a matter of definition, how to name the policies predictably causing enhanced mortality. Apart from alcohol, limited availability of modern health care is an obvious cause of the relatively low life expectancy in Russia (8). It should be mentioned in conclusion that drinking of wine has decreased since the Soviet time, whereas beer has increased (14). Soviet-time sorts of cheap fortified wine have largely disappeared; while new inexpensive domestic and imported products are appearing, that can contribute to the increase in wine consumption in future. Their quality is however unstable; and surrogates can be encountered now as before. Authorities should take measurements to prevent the sales of wine surrogates, other poor-quality drinks and counterfeit alcoholic beverages. However, manufacturing of some popular Soviet-time fortified wines can be resumed under a condition of strict adherence to the original formulas.

Kotlas (Arkhangelsk region a northern town) is known to be a home of numerous alcohol users and abusers, among them former prisoners, and also some people compulsorily resettled from Moscow during the 1970-80s. Today the situation seems to be improving, heavy binge drinking being visibly in decline. It is surrogate in the bottles shown on this image. Portwein 72 had been in expensive red fortified wine with acceptable quality during the Soviet time. It has disappeared; and the popular label is used for selling of surrogates. "Port wine" 777 appeared later in the 1980s and has had poor quality from the beginning (15-17).
